# Delivery of home-based postpartum contraception in rural Guatemalan women: a cluster-randomized trial protocol

**DOI:** 10.1186/s13063-019-3735-3

**Published:** 2019-11-21

**Authors:** Margo S. Harrison, Saskia Bunge-Montes, Claudia Rivera, Andrea Jimenez-Zambrano, Gretchen Heinrichs, Sharon Scarbro, Elizabeth Juarez-Colunga, Antonio Bolanos, Edwin Asturias, Stephen Berman, Jeanelle Sheeder

**Affiliations:** 1 University of Colorado, Mail Stop B198-2, Academic Office 1, 12631 E. 17th Avenue, Rm 4211, Aurora, Denver, CO 80045 USA; 2 Fundación para la Salud Integral de los Guatemaltecos (FSIG), Quetzaltenango, Guatemala; 3 Denver Health, Denver, CO USA

**Keywords:** Postpartum contraception, Long-acting reversible contraceptives, Implant, Nursing, Community programming, Cluster-randomized trial

## Abstract

**Background:**

Postpartum contraception is important to prevent unintended and closely spaced pregnancies following childbirth.

**Methods:**

This study is a cluster-randomized trial of communities in rural Guatemala where women receive ante- and postnatal care through a community-based nursing program. When nurses visit women for their postpartum visit in the intervention clusters, instead of providing only routine care that includes postpartum contraceptive education and counseling, the nurses will also bring a range of barrier, short-acting, and long-acting contraceptives that will be offered and administered in the home setting, after routine clinical care is provided.

**Discussion:**

A barrier to postpartum contraception is access to medications and devices. Our study removes some access barriers (distance, time, cost) by providing contraception in the home. We also trained community nurses to place implants, which are a type of long-acting reversible contraceptive method that was previously only available in the closest town which is about an hour away by vehicular travel. Therefore, our study examines how home-based delivery of routinely available contraceptives and the less routinely available implant may be associated with increased uptake of postpartum contraception within 3 months of childbirth. The potential implications of this study include that nurses may be able to be trained to safely provide contraceptives, including placing implants, in the home setting, and provision of home-based contraception may be an effective way of delivering an evidence-based intervention for preventing unintended and closely spaced pregnancies in the postpartum period.

**Trial registration:**

Clinicaltrials.gov, NCT04005391. Retrospectively registered on 1 July 2019.

## Introduction

### Background and rationale

Postpartum contraception is important for preventing unintended and closely spaced pregnancies following childbirth as well as to avoid future pregnancy in mothers who have achieved their desired family size [1]. Proper pregnancy spacing can prevent maternal and perinatal morbidity and mortality [1]. Globally, there is a significant unmet need for postpartum contraception [1]. In Guatemala, one prior study in a community close to our study site suggests about two-thirds of women reportedly have an unmet need for postpartum contraception [2].

Historically, in our community of interest, which is in the rural southwest corner of Guatemala bordering Mexico, a large majority (88%) of a convenience sample of women in our community-based care program self-reported postpartum contraceptive use. However, 72% of these women were using injectable contraceptives, which are considered to be short-acting and are less effective at preventing unintended and closely spaced pregnancies; the second most common method was sterilization (21%). For the remaining women who did not seek sterilization or injectable contraceptives, 0.5% of them reportedly used contraceptive pills, 0.5% used condoms, 0.5% used lactational amenorrhea, and 1.6% reportedly relied on natural family planning. Less than 4% of women were using long-acting reversible contraceptives (intrauterine devices or implants), which are more effective at preventing unintended and closely spaced pregnancies than injectable contraceptives (Harrison: Postpartum contraception in rural Guatemala: results of a quality improvement database, unpublished). The providers in the community had not been trained in implant and intrauterine device placement, so the closest place to have a device placed was the most proximate town to our communities, which is an hour away by vehicular travel. Additional historical barriers to the use of family planning discovered through qualitative research in our study communities included knowledge, access to methods, fear of adverse events, and a woman garnering her partner’s approval [3]. Ideas from the community women for future educational programming included teaching about how birth control methods work, how to talk to partners about birth spacing, and myth debunking, and they requested the location and time of teaching to occur at peripartum and pediatric visits [3].

Therefore, based on these preliminary data, our prospective research question is: if we train the community-based nurses to place contraceptive implants, and offer women placement of these implants in the home at their postpartum visit (among other routinely available contraceptive methods), will this service increase the uptake of contraceptive implants in our communities of interest?

### Specific objectives or hypotheses

The specific objective of our study is to observe whether home delivery of the contraceptive implant increases utilization of the device above the baseline rate of 3.2% in this population where the use of postpartum contraception is historically common among women. Our hypothesis, based on published data from other settings, is that if women receive proper counseling about all contraceptive methods, the uptake of long-acting reversible contraceptives (which in this study includes only the contraceptive implant) will be about 11% for contraceptive implants [4].

Our secondary objectives are to observe if the home-based contraceptive delivery intervention featured in our study is associated with overall increased contraceptive uptake, continuation, and satisfaction in the intervention compared to the control clusters. We hypothesize that with increased uptake of the implant we will see higher continuation rates and contraceptive satisfaction in the intervention clusters. We also hypothesize that increased contraceptive uptake, continuation, and satisfaction may be associated with reduced short-interval, repeat pregnancy rates, and so we also aim to survey participants on their pregnancy status at enrollment and 3 and 12 months after enrollment.

### Trial design

Our protocol features an interventional, cluster-randomized, unmasked, parallel group trial design. Our eight study communities were randomized in a 1:1 allocation ratio to either the intervention or control arms of the study, and our study is designed on a superiority framework.

## Methods: participants, interventions, and outcomes

### Study setting

The study setting is in the home of women enrolled in the “Madres Sanas” community-based nursing program offered by the Fundación para la Salud Integral de los Guatemaltecos (FSIG) [5]. FSIG supports a community-based clinic called the Center for Human Development in a region of Guatemala informally referred to as the southwest Trifinio [5]. This region is at the intersection of the boundaries of three departments in Guatemala, and as such no single department takes responsibility for the health of the migrant workers that reside there [5]. This impoverished area has a population of around 25,000 people and experiences poor pregnancy outcomes [5]. This is why the University of Colorado Center for Global Health, in partnership with AgroAmerica, built the Center for Human Development clinic and initiated the community-based maternal and perinatal care programs, called Madres Sanas and Ninos Sanos, respectively [5].

**Fig. 1:**
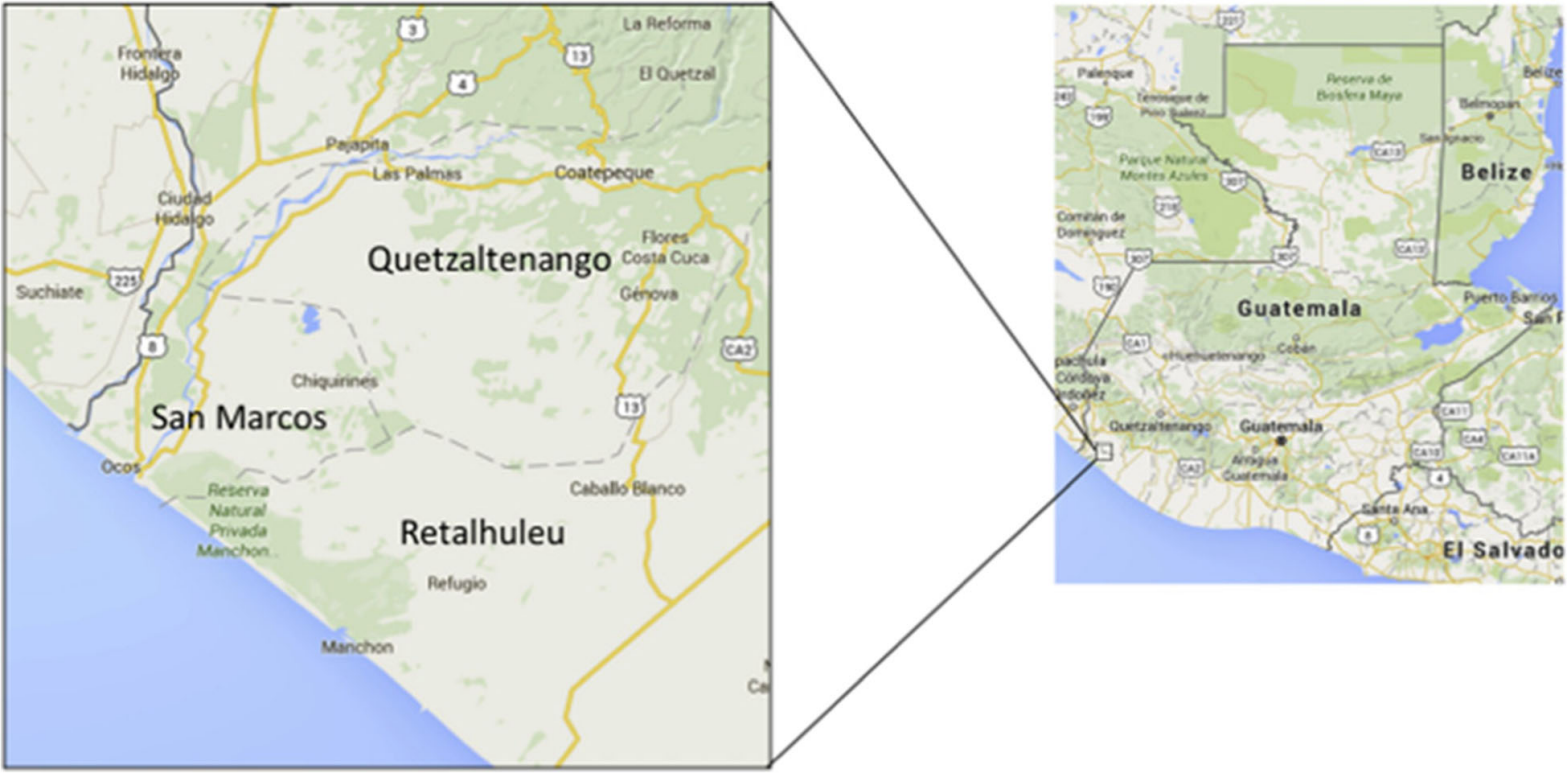
Map of the southwest Trifinio area of Guatemala where our study is being executed

Madres Sanas is executed by teams of Guatemalan community nurses. The nurse teams are comprised of two nurses who are responsible for a segment of the communities in the region. There are ten communities in the Madres Sanas program that are combined into eight clusters; our biostatistician did this in order to achieve similar cluster sizes, determined according to the number of births by community in 2017. Because our study enrolls women at their final Madres Sanas visit, which is a postpartum visit that occurs about 40 days after delivery, the cluster size was based on delivery volume of the communities. The nurse teams are assigned by the nursing supervisor to their respective communities. They drive auto rickshaws provided by the Center for Human Development out to the communities to conduct their home visits, which includes four antenatal visits and two postpartum visits. During the visits the nurses both provide clinical care and collect quality improvement and research data, and as such serve a dual function in their role. As noted, this study takes place at the final Madres Sanas visit, which occurs 40 days after delivery. Routine clinical care, including postpartum contraceptive education, culminates at this time, although counseling on postpartum contraception begins at the initial enrollment visit during pregnancy. After routine clinical care is provided, the nurses offer enrollment in our study. Figure 1 shows a map of the study communities [5].

### Eligibility criteria

All women capable of providing informed consent who participate in the Madres Sanas program and have a 40-day postpartum visit are eligible to enroll in the study if they are between the ages of 15–35 years and have not already started a contraceptive method. Those unable or unwilling to provide consent are excluded.

### Interventions

All study activities and procedures begin at the 40-day postpartum visit, which is the sixth and final scheduled visit of the Madres Sanas program. At this visit the community nurses perform routine maternal and neonatal clinical care, which includes, amongst other activities, final counseling and education about postpartum contraception. In intervention clusters, nurse teams bring a kit with them to the visit. After the visit they describe the study and offer consent. Once a woman has discussed the risks, benefits, and alternatives with her providers and determines she wishes to participate in the study, she signs the consent form and the nurses offer her condoms, contraceptive pills, a medroxyprogesterone contraceptive injection, or the levonorgestrel implant. The kit is stocked with 10 condoms (Vive Amor®), one pack of pills (Segura Plus®), one syringe of medroxyprogesterone (Cyclofem®), and one implant (Jadelle®) for each postpartum visit planned for that day. It also contains all the necessary materials to place the implant or administer the injection under sterile conditions, such as alcohol swabs and sterile gloves and so forth.

All contraceptives are purchased using study funds and are sourced from a local provider of contraceptive medications and devices. All contraceptives are routinely available and approved for distribution in Guatemala. Because these are routinely available medications and we are not testing their effectiveness as contraceptives, there are no criteria for discontinuing or modifying allocated interventions for a given trial participant (such as changing the drug dosing). Women are, however, screened for contraindications to the contraceptive methods provided using the Medical Eligibility Criteria [6]. The nurses have a laminated Spanish language version of the eligibility criteria chart included in their kit for use during study enrollment. As this is a pragmatic trial, there are no restrictions on care and interventions that are permitted or prohibited during the trial. For example, if a participant did not initiate a contraceptive method at the Madres Sanas nurse visit and wishes to, she can seek the method in the community. Conversely, if she chose a method and opted for the implant but does not like it, she is free to remove it at any time. The nurses will either remove it in the home setting or advise the woman to present to the Center for Human Development where they can remove it and potentially initiate a new method. The initial contraceptive provided in the study setting is free, but any contraceptives sought or utilized after the study enrollment visit is the woman’s responsibility to locate and finance. Study contraceptives are only provided in the home setting by the nurses at the enrollment visit and are not available or offered at any subsequent visit.

### Outcomes

Our primary outcome is the proportion of women using the contraceptive implant at 3 months after enrollment in the intervention clusters as compared to the control clusters. We are planning a difference in differences analysis of proportions. Our secondary outcomes are to also compare overall contraceptive uptake, continuation, satisfaction, and pregnancy rates between study arms. All these data are collected by maternal self-report through enrollment, 3-month, and 12-month surveys.

The clinical relevance of our primary outcome is that we have the potential to reduce unintended and short-interval pregnancies by increasing utilization of a highly effective method of postpartum contraception (the implant). The clinical relevance of monitoring continuation, satisfaction, and pregnancy rates is to observe the acceptability of the implants in the community; for example, it is possible that we find a significant primary outcome we also find that women did not like the implant and took it out before 3 months. This will provide important information to contextualize our primary outcome.

#### Primary outcome

The primary outcome of this study is use of the contraceptive implant at 3 months postpartum.

Postpartum implant use will be measured as the proportion of women in each arm of the trial who self-report using a contraceptive implant when they complete their 3-month survey. The timepoint for this will be 3 months following enrollment in the study.

#### Key secondary outcomes

Postpartum contraceptive use will be measured as the proportion of women in each arm of the trial who self-report using a contraceptive method when they complete their 3-month survey. The timepoint for this will be 3 months following enrollment in the study.

Postpartum contraceptive continuation will be measured as the proportion of women in each arm of the trial who self-report using a contraceptive method when they complete their 12-month survey. The timepoint for this will be 3 and 12 months following enrollment in the study.

Postpartum contraceptive satisfaction will be measured as the proportion of women in each arm of the trial who self-report satisfaction (if they are using a contraceptive method) with that method when they complete their 3-month and 12-month survey. The timepoint for this will be 3 and 12 months following enrollment in the study.

Short-interval pregnancy will be measured as the proportion of women in each arm of the trial who self-report repeat pregnancy when they complete their enrollment, 3-month, and 12-month surveys. The timepoint for this will be at enrollment and 3 and 12 months following enrollment in the study.

### Participant timeline

**Fig. 2:**
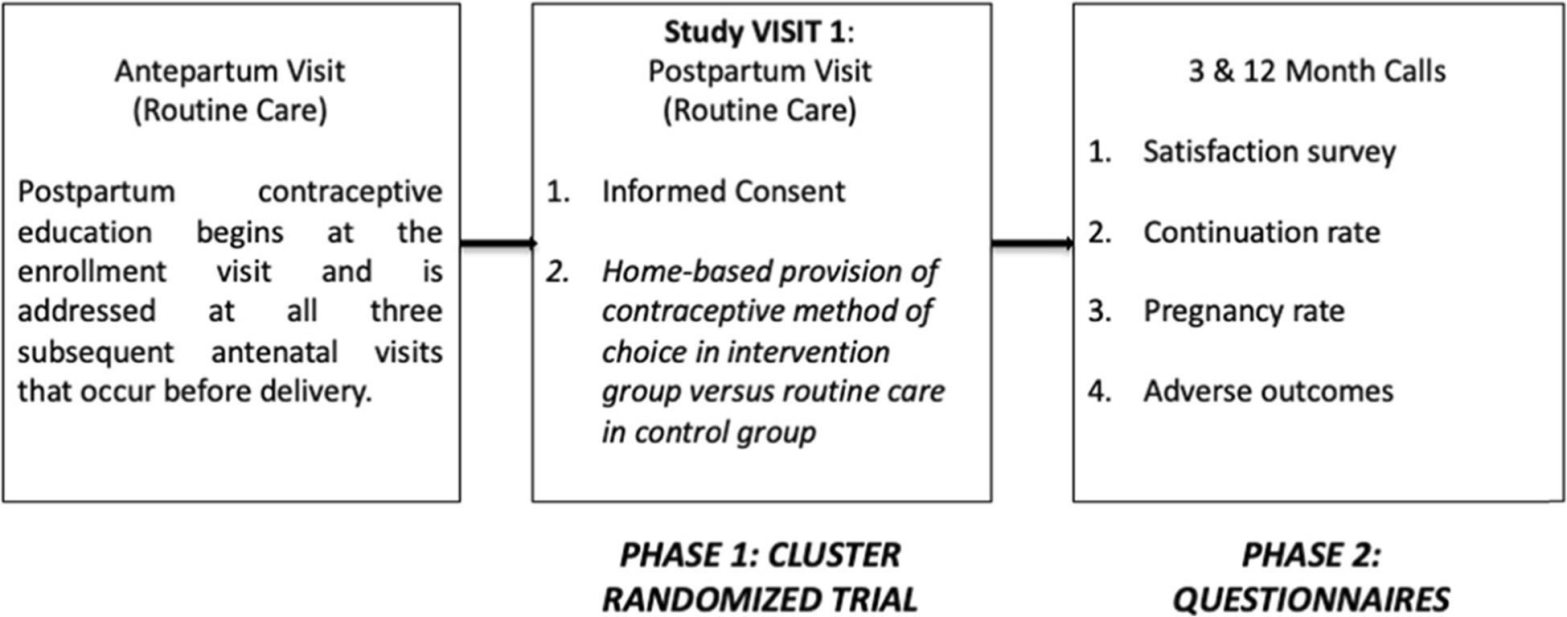
Study activities

Women are enrolled as part of their routine postpartum visit in the Madres Sanas program that occurs about 40 days after a delivery. Three months after study enrollment, women are either called or visited in their home to complete a survey regarding their current contraceptive use, potential contraceptive continuation, satisfaction with any contraceptive they might be using, and whether or not they have been pregnant since the delivery of their last child. Similar questions are asked 12 months after study enrollment to observe our secondary outcomes; after the 12-month survey is conducted the study activities are considered complete. Figure 2 describes the study activities.

### Sample size

**Fig. 3:**
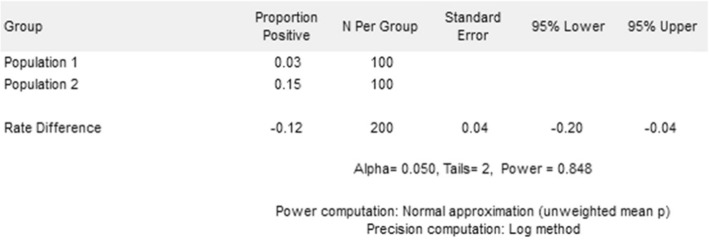
Sample size calculation

Based on previously collected data, we expect that during the timeframe of our study around 260 women will meet the eligibility criteria over the course of 1 year. This study, with 200 women enrolled (100 in intervention clusters and 100 in control clusters) will be powered to detect a change in Jadelle® uptake rates from 3% to 15% at 85% power and 5% significance, with an intraclass correlation of 2% (Fig. 3).

### Recruitment

As part of our educational efforts within the Madres Sanas program we use flipcharts. These flipcharts are spiral-bound, laminated compilations of images that represent health-care concepts. As some women in our community are not literate and because many people are visual learners, the nurses bring flipcharts that are specific to the visit they are executing to help guide them and their patients through the educational sessions that are a component of every care session. These flipcharts have been shown to be a great way to both structure the visits and to ensure that important concepts are covered, and they have worked well at imparting information. As such, our main strategy for achieving adequate participant enrollment was to create a study flipchart and an informed consent flipchart to illustrate the main concepts of the study and to help the nurses standardize and ensure a proper informed consent process.

The nurses also bring contraceptive educational materials with them to antepartum visits. They introduce the idea of optimizing child health by optimizing pregnancy spacing. The nurses have a model arm with an implant in it that women can palpate, contraceptive pill packs, sample intrauterine devices, and condoms, so that over the course of their antenatal care women are able to touch and visualize different contraceptive methods. This allows them to become more comfortable with the concept of postpartum contraception/pregnancy spacing and the various options; thus, when the study is offered at the end of their Madres Sanas experience, they have some familiarity and health literacy with respect to the options offered as part of the study. Our final strategy for achieving adequate participant enrollment to reach our target sample size is to offer enrollment to all women at their Madres Sanas postpartum visit who meet our inclusion and exclusion criteria.

## Methods: assignment of interventions

### Sequence generation

The first step, as noted previously, was to divide our Madres Sanas communities into study clusters. We were able to obtain historical data about the number of enrollees/births by community to get a sense of the volume of births in each area. We combined some smaller communities into larger study clusters in order to obtain an expected birth rate of about 100 births per nurse team (nurse teams have varying numbers of clusters) per year for a total of about 300 expected births in 1 year. Once the clusters were assigned by expected birth volume, which we expect to translate to eventual postpartum visits, the allocation sequence was generated.

### Allocation and concealment mechanism

The initial allocation sequence was generated by our data analyst using SAS to assign the clusters to either the intervention or the control arm of the trial. Once the nurses were educated about the study and understood all study procedures and activities, they were informed about the cluster assignment. One of the nurse teams was not assigned an intervention group and the nursing supervisor requested that each nurse team have an intervention and a control group. As such, the allocation sequence was rerun to accommodate the real-world constraints of the study to appease the study staff in order to proceed with study activities; this will be addressed as a limitation of our study when we publish our results. The communities, assigned to clusters, are described with their nurse team (as indicated by team color) in Table 1.

**Table 1:**
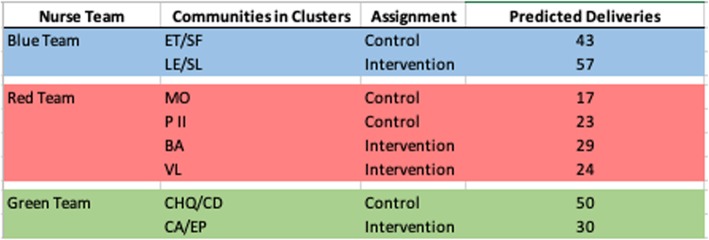
Communities combined into clusters and nurse teams (by color) with predicted deliveries per cluster

*EL/SF* El Troje/Sante Fe, *LE/SL* Los Encuentros/San Luis, *MO* Morenas, *PII* Palmar II, *BA* Barillas, *VL* Valle Lirio, *CHQ/CD* Chiquirines/Colonia Diaz, *CA/EP* Carizales/El Pomal

### Implementation

It is the responsibility of the nurse to enroll patients in the study and to only offer home-based contraceptives to women living in intervention clusters. If the nurses incorrectly offer the intervention in control clusters this will bias our study towards the null hypothesis of there being no difference in the uptake of the contraceptive implant.

### Blinding

Our study did not involve any blinding procedures; neither the nurses nor the participants were blinded to their assignment, so there was no unblinding necessary.

## Methods: data collection, management, and analysis

### Data collection methods

**Fig. 4:**
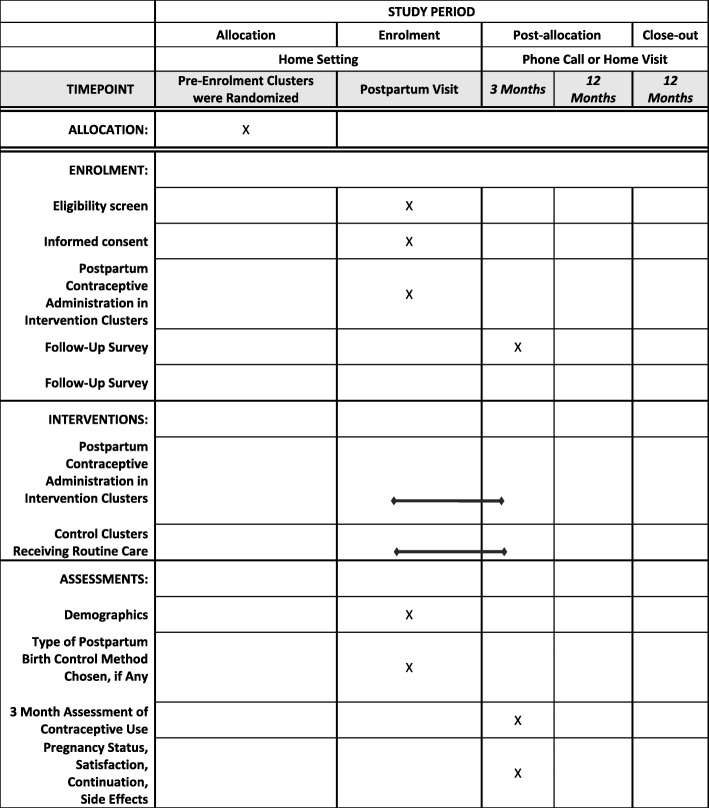
Schedule of enrolment, interventions, and assessments

We have quality improvement data prior to study initiation that was collected from June 2017 to September 2018 by the Madres Sanas community nurses. This database includes antepartum, intrapartum, and postpartum quality improvement data collected by our community nurses during routine antenatal and postnatal care visits. The data are collected on tablets and transmitted through the Research Electronic Data Capture (REDCap) application [7]. REDCap is a secure, HIPAA-compliant web-based application designed for data collection for research studies [7]. It provides an easy-to-use data entry system with data validation, the ability to import data from external sources (such as Guatemala), automated exports to statistical software, audit trails, branching logic and calculations, and sophisticated tools for building and managing online surveys [7]. As the community nurses have been using this software for years, our study links to the Madres Sanas dataset but involves separate forms in a separate REDCap database. The nurses will use REDCap and the same methods to collect enrollment and follow-up data on participants as they do during routine visits to collect general pregnancy outcomes data for our quality improvement database; these study data will be transmitted to password-protected servers at the University of Colorado. There are cluster-specific REDCap forms that are collected on enrollment, at 3 months, and at 12 months following enrollment. These surveys are included in Additional file 1. The schedule of enrolment, interventions, and assessments are shown in Fig. 4.

### Data management

Our study supports a data manager who reviews the study data on a biweekly basis for inconsistencies and provides a study dashboard of study progress in terms of enrollment, retention, and follow-up. Our plans to promote participant retention and to complete follow-up include having the nurse teams conduct the 3- and 12-month surveys on their own enrollees from their own communities. In this way, the personal relationship that was built during the antepartum and postpartum care programming is continued after enrollment in an effort to provide continuity of the relationship through study activities.

### Statistical methods

To analyze our primary outcome we are planning a difference in differences analysis of the proportion of women actively using a contraceptive implant 3 months after enrollment in intervention clusters as compared to control clusters. We plan to provide an unadjusted analysis and an adjusted analysis for any cluster characteristics that are imbalanced between the two study arms. Once our primary outcome is assessed, we will likely use multivariate modeling to determine characteristics of women in each study arm who used any postpartum contraception within 3 months of delivery as compared to those who did not, in both adjusted and unadjusted analysis. We plan to use descriptive statistics to compare our other secondary outcomes between study arms (continuation and satisfaction). Finally, we will likely perform survival analysis of time to repeat pregnancy by study arm to observe if there is any statistically significant difference in the time to repeat pregnancy of women who became pregnant by 12 months in the study groups. We plan to use intention-to-treat in our analysis.

## Methods: monitoring

### Data monitoring

This study does not have a data monitoring committee because we are not testing the safety or efficacy of a new intervention but rather the association of increasing access to the postpartum contraceptive implant with uptake of the device. Do not have any predefined stopping guidelines. No one will have access to any interim results that might influence a decision to terminate the trial early (Additional file 2).

### Harms

The Center for Human Development has a close relationship with the community it serves. It meets regularly with community leaders from each community that comprise the Community Advisory Board, which approved the study before its execution. The community leaders permitted the Center for Human Development to develop the community-based nursing programs and help set the priorities for care in the programs. They meet monthly with the Center for Human Development leadership to discuss issues related to the provision of care in the community. In addition to the Community Advisory Board as a mechanism for communication between these clinical and research partners, the community nurses themselves serve as a genuine link between the community and FSIG/the Center for Human Development. The nurses are in the communities 5 days a week providing preventative care and managing pregnancy and neonatal/early childhood complications. As such, our main plans for collecting, assessing, reporting, and managing solicited and spontaneous reported adverse events or other unintended effects of our trial intervention depend on the nurses, the Community Advisory Board, and the women themselves. We will not be monitoring specifically for adverse effects of study medications as they are not themselves under study. Therefore, we depend on women to contact their community nurse and, if they do not feel comfortable, their community leadership regarding any adverse events they are experiencing. The nurses share their work cell phone numbers and patients call them and the nursing supervisor frequently. Patients also know that the Center for Human Development clinic is open to provide care to them at any time. In this way, we hope that the community safety net established through trust and partnership between the Center for Human Development and the community will help with finding and managing adverse events during the study. There are also standard operating procedures for the study staff to follow if any of these adverse events occur.

### Auditing

The Principal Investigator (PI) and the Senior Foreign Investigator (SFI) are responsible for auditing trial conduct in person, while the data manager audits the actual data being entered on a biweekly basis. Our team meets as a group to discuss study activities every other week, and data issues and inconsistencies are often addressed at that time. Between the PI, SFI, and other co-investigators on the study, site visits are made every 1 to 3 months to observe study activities and to provide audit and feedback on the consent process and data entry. There are no planned procedures for an independent audit of trial conduct.

## Ethics and dissemination

### Research ethics approval

This study protocol, the data collection forms, and the consent form were approved by both the Colorado Multiple Institutional Review Board and their International Research Advisory Committee (COMIRB no. 17–1314) in the USA and by the Instituto de Nurtición de Centro América y Panamá (INCAP) in Guatemala (CIE-REV 076/2018), as well as by the Community Advisory Board. Both ethics review committees are providing ongoing review of the study as it is being conducted.

### Protocol amendments

Any protocol modifications will be communicated to COMIRB and INCAP at the time of modification and again on annual review according to the procedures of the ethics review committees.

### Consent

The community nurses who double as the study staff for this project will obtain informed consent and assent from potential trial participants or authorized surrogates using the flipchart and the informed consent documentation, as previously described. The consent includes language about the use of patient data for analysis but no patient identifiers except study cluster are of relevance to the primary outcome. No biological specimens are being collected that might be used for ancillary studies. Personal information about the participants is collected on study forms and stored in REDCap, as previously described. We plan to and have ethics approval to link our forms by the participants’ Madres Sanas identification number to use routinely collected quality improvement sociodemographic and pregnancy data to describe the women included in our study sample.

### Confidentiality

The data is stored on a password-protected server at the University of Colorado. No one will be able to access the data other than team members listed on the institutional review board application, and data access will be monitored by the data manager. After the trial is complete data will be kept until the ethics approvals are no longer able to be renewed, at which point the data will be destroyed.

### Access to data

The PI, SFI, and data manager will have access to the final trial dataset, as outlined in the institutional review board approval. If other members of the team wish to access the data we will have to pursue an ethical review addendum, but we have no plans to broaden access to the dataset at this time. We have no contractual agreements specific to this dataset, but the relationship between the University of Colorado and FSIG is mediated by a memorandum of understanding.

### Ancillary and post-trial care

Patients have access to the community nurses and the clinic at the Center for Human Development for ancillary and post-trial care. Care related to any adverse outcomes of any contraceptive use will be covered by study funds, but routine contraceptive management and care will not be covered under study protocols. We do not intend to compensate participants who suffer harm from the trial beyond providing treatment for adverse outcomes of trial participation.

### Dissemination policy

We plan to communicate trial results to the Community Advisory Board as a way of communicating back to the community and the study participants as we do not have a convenient way to provide results to the women such as a mailing or email address. We intend to disseminate the results to health-care professionals and the public via publication of abstracts, manuscripts, and oral presentations at Guatemalan and American obstetric and gynecologic conferences. We do not currently have any publication restrictions. Authorship will include the same members of the team and authorship as the protocol and we do not intend to use professional writers.

## Trial status

This is protocol version v05-Oct-2017. The date of first enrollment was 23 October 2018. The study is anticipated to be fully enrolled by 31 December 2019, and we anticipate our final study data to be collected by 31 December 2020.

## Supplementary information

**Additional file 1.** Data collection forms.

**Additional file 2.** SPIRIT checklist.

## Data Availability

We intend to publish the protocol but have no plans to provide the participant-level dataset at this time. As this study is funded by the National Institutes of Health, the dataset may be required to become publicly available at some point in the future. Statistical code will be provided by specific request.

## Abbreviations

COMIRB: Colorado Multiple Institutional Review Board and their International Research Advisory Committee
FSIG: Fundación para la Salud Integral de los Guatemaltecos
INCAP: Instituto de Nurtición de Centro América y Panamá
PI: Principal Investigator
REDCap: Research Electronic Data Capture
SFI: Senior Foreign Investigator
