# Supplementary material for: Delivery of home-based postpartum contraception in rural Guatemalan women: a cluster-randomized trial protocol
Source: Trials. 2019 Nov 21;20:639. doi: 10.1186/s13063-019-3735-3 (PMC6869182; doi:10.1186/s13063-019-3735-3)
Supplement: Supplementary file 1 — Additional file 1. Data collection forms. [file 13063_2019_3735_MOESM1_ESM.pdf]

Abordaje

Código de Identificación

____

Completar esta hoja de registro para TODOS los sujetos que sean abordados para este estudio. (Se llena para mujeres que no quieren participar y también para mujeres que no puede participar porque no cumple con los criterios).

Equipo

- ◯ Azul
- ◯ Rojo
- ◯ Amarillo

Fecha de Abordaje

____

¿Es elegible?

- ◯ Si
- ◯ No

Si no es elegible, explique ¿por que no?

____

¿Quiere participar?

- ◯ Yes
- ◯ No

Si no quiere participar, explique ¿por que no?

____

Iniciales

____

Encuesta Inicial

Fecha

____

Consentimiento

Consentimiento completado

◯ Si
◯ No

Si no, por que?

____

Foto del consentimiento completo

Nombre

____

¿Cuantos partos ha tenido?

____
((Esto incluye bebes nacidos vivos, bebes que han fallecido despues del parto y bebes que han muerto intrautero))

Fecha de Parto

____
(Favor de eligir la fecha del parto MAS RECIENTE)

Comunidad

◯ Morenas
◯ Valle Lirio
◯ Palmar II
◯ Barillas
◯ Carrizales
◯ Chiquirines
◯ Chiquirines- Colonia Diaz
◯ Los Encuentros
◯ Los Encuentros- San Luis
◯ Pomal
◯ Santa Fe
◯ El Troje
(REVISAR SI ES INTERVENCIÓN O CONTROL)

Resultado de la prueba de embarazo urinaria:
NO EMPIECE EL ANTICONCEPTIVO SI ESTÁ EMBARAZADA

☐ Negativo
☐ Positivo
☐ No Aplica

PARA LA ENFERMERA: Si usted administró o fue a dejar/distribuyó el anticonceptivo a la paciente, por favor escriba el número de lote, expiración, y el resultado de la prueba de embarazo.

¿Qué método anticonceptivo escogió usted hoy?	◯ Ningunos de los métodos ofrecidos<br>◯ Condones<br>◯ Pastillas<br>◯ Inyección<br>◯ Implante<br>((Se le ofreció a la paciente condones, pastillas, depo, y jadelle en la casa))
Número de Lote	__________________________
Fecha de expiración	__________________________
¿Por qué escogió este/estos métodos?	☐ Lo he usado antes<br>☐ Otra gente en mi comunidad los ha usado y les ha gustado<br>☐ Creo que es la mejor opción que la enfermera ofreció<br>☐ Puedo pagar por este método cuando lo necesite otra vez<br>☐ Otro<br>((marque todos los que apliquen))
Si escogió otro, explique	__________________________
¿Qué método anticonceptivo está planeando utilizar?	☐ No estoy planeando en empezar un método anticonceptivo<br>☐ Estoy planeando en abstenerme<br>☐ Estoy planeando usar el método natural de familia/método rítmico<br>☐ Estoy planeando usar amenorrea de la lactancia/ dar de mamar<br>☐ Estoy planeando usar condones pero no quería empezarlos en la casa hoy.<br>☐ Estoy planeando usar las pastillas pero no las quería empezarlas en la casa hoy.<br>☐ Estoy planeando usar la inyección pero no quería empezarla en la casa hoy.<br>☐ Estoy planeando usar el implante pero no quería empezarlo hoy.<br>☐ Estoy planeando usar la T de cobre<br>☐ Estoy planeando usar operación/ligadura<br>☐ Mi esposo está planeando hacerse una vasectomía<br>☐ Mi esposo se hizo la vasectomía<br>☐ No estoy segura/ No sé
¿En dónde planea conseguir este/estos métodos si no escogió un método anticonceptivo hoy?	☐ Clínica Privada<br>☐ Puesto de Salud<br>☐ Farmacia<br>☐ Hospital<br>☐ No sé<br>☐ Otro<br>((marque todos que apliquen))
Si escogió otro, explique	__________________________

¿En dónde planea conseguir este/estos métodos?	☐ Clínica Privada<br>☐ Puesto de Salud<br>☐ Farmacia<br>☐ Hospital<br>☐ No sé<br>☐ Otro<br>((marque todos que apliquen))
Si escogió otro, explique	__________________________________
Si la paciente escogió "No estoy planeando empezar un método de contracepción", por favor pregunte, ¿por qué?	☐ Quiero quedar embarazada<br>☐ No tengo una pareja entonces no necesito un método anticonceptivo.<br>☐ Mi pareja no está de acuerdo que use un método anticonceptivo.<br>☐ Tengo miedo de los efectos secundarios<br>☐ No quiero empezar un método anticonceptivo porque no voy a poder continuarlo por problemas de tiempo, costo, o transporte.<br>☐ No sé<br>☐ Otro
Si escogió otro motivo por el cual no esta usando un método de contracepción, explique el motivo	__________________________________
Si escogió que tiene miedo de los efectos secundarios, ¿cuáles son esos efectos secundarios?	__________________________________
Si la paciente no escogió el implante, pregúntele, ¿por qué no escogió el implante?	☐ Quiero quedar embarazada<br>☐ No tengo una pareja entonces no necesito un método anticonceptivo.<br>☐ He usado el método que escogí antes, y lo quería usar ahorita, otra vez.<br>☐ Tengo miedo de los efectos secundarios<br>☐ He oído malas cosas sobre el implante<br>☐ Tengo miedo que me pongan el implante en el brazo<br>☐ Tengo miedo que me pongan el implante en la casa<br>☐ No quiero que me quiten el implante en el futuro<br>☐ No sé<br>☐ Otro
Si escogió que tiene miedo de los efectos secundarios del implante, ¿cuáles son esos efectos secundarios?	__________________________________
Si ha oido cosas malas, ¿qué ha oído?:	__________________________________
Si escogió otro motivo por el cual no usa el implante, explique el motivo	__________________________________
Si le ofreciéramos un dispositivo intrauterino (DIU) en su casa durante una visita, ¿estaría interesada en recibirlo?	◯ Sí<br>◯ No<br>◯ No sé<br>◯ Otro
Si escogió otro motivo, explique	__________________________________

Llamada de Seguimiento- 3 Meses (Grupo de Intervencion CON METODO)

En su visita de postparto la enfermera le dio ¿todavía sigue usando este método?	◯ Si<br>◯ No<br>((muestre el método que la paciente escogió en la primera pregunta de la encuesta Int de PPV, posiblemente: pastillas, condones, inyección, o implante))
¿Qué tan satisfecha esta con este método?	◯ Muy satisfecha<br>◯ Un poco satisfecha<br>◯ Un poco insatisfecha<br>◯ Muy insatisfecha
Por favor, discúlpese por esta insatisfacción y pregunte ¿por qué?	☐ El medicamento causa efectos secundarios<br>☐ Es muy cara<br>☐ Es muy difícil de obtener<br>☐ No está siempre disponible<br>☐ Otro<br>((Marque todos los que apliquen))
Si escogió otro, explique	__________________________________
Si escogió que el medicamento causó efectos secundarios, ¿cuáles son esos efectos secundarios?	__________________________________
¿Por qué descontinuó el método?	☐ No me gustaban los efectos secundarios<br>☐ Quería quedar embarazada<br>☐ No podía pagar la continuación del método<br>☐ No podía pagar el transporte para obtener el método<br>☐ El método no estaba disponible cuando lo fui a obtener<br>☐ No tengo una pareja entonces no quería continuar el método<br>☐ Mi pareja me pidió que dejara de usar el método<br>☐ No sé<br>☐ Otro<br>((Marque todos los que apliquen))
Si escogió otro ¿por qué otro motivo descontinuó el método?	__________________________________
¿Empezó un método nuevo?	◯ Si<br>◯ No

¿Qué método está utilizando?	☐ Abstinencia<br>☐ Método natural de familia/método rítmico<br>☐ Amenorrea de la lactancia/ dar de mamar<br>☐ Condones<br>☐ Pastillas<br>☐ Inyección<br>☐ Implante<br>☐ Dispositivo intrauterino (DIU)<br>☐ Operación/Ligadura<br>☐ Mi esposo se hizo la vasectomía<br>((Marque todos los que apliquen))
Si "no", ¿por qué?	☐ Quiero quedar embarazada<br>☐ No tengo una pareja entonces no necesito un método anticonceptivo.<br>☐ Mi pareja no esta de acuerdo que use un método anticonceptivo.<br>☐ Tengo miedo de los efectos secundarios<br>☐ No quiero empezar un método anticonceptivo porque no voy a poder continuarlo por problemas de tiempo, costo, y transporte.<br>☐ No sé<br>☐ Otro<br>((Marque todos los que apliquen))
Si escogió otro motivo por el cual no esta usando un método de contracepción, explique el motivo	__________________________________
Si escogió que tiene miedo de los efectos secundarios, ¿cuáles son esos efectos secundarios?	__________________________________
Desde que dio a luz, ¿ha vuelto a quedar embarazada?	◯ Sí<br>◯ No<br>◯ No sé
¿Qué pasó con el embarazo?	◯ Aborto espontáneo<br>◯ Terminación del embarazo<br>◯ Actualmente, estoy embarazada

ENFERMERA: SI LA PACIENTE ESTÁ ACTUALMETNE EMBARAZADA, DEBERÍA ADVERTIRLE QUE PARE SU MÉTODO ANTICONCEPTIVO SI LO SIGUE USANDO Y QUE SE INSCRIBA EN MADRES SANAS.

Llamada de Seguimiento- 3 Meses (Grupo de Intervencion SIN METODO)

¿Ha empezado un método anticonceptivo desde que dió a luz?	◯ Si<br>◯ No
¿Qué método está utilizando?	☐ Abstinencia<br>☐ Método natural de familia/método rítmico<br>☐ Amenorrea de la lactancia/ dar de mamar<br>☐ Condones<br>☐ Pastillas<br>☐ Inyección<br>☐ Implante<br>☐ Dispositivo intrauterino (DIU)<br>☐ Operación/Ligadura<br>☐ Mi esposo se hizo la vasectomía<br>((Marque todos los que apliquen))
¿Qué tan satisfecha esta con este método?	◯ Muy satisfecha<br>◯ Un poco satisfecha<br>◯ Un poco insatisfecha<br>◯ Muy insatisfecha
¿Por qué esta insatisfacción con este método?	☐ El medicamento causa efectos secundarios<br>☐ Es muy cara<br>☐ Es muy difícil de obtener<br>☐ No está siempre disponible<br>☐ Otro
Si escogió otro, explique	__________________________________
Si escogió que el medicamento causó efectos secundarios, ¿cuáles son esos efectos secundarios?	__________________________________
Si "no", ¿por qué?	☐ Quiero quedar embarazada<br>☐ No tengo una pareja entonces no necesito un método anticonceptivo.<br>☐ Mi pareja no esta de acuerdo que use un método anticonceptivo.<br>☐ Tengo miedo de los efectos secundarios<br>☐ No quiero empezar un método anticonceptivo porque no voy a poder continuarlo por problemas de tiempo, costo, y transporte.<br>☐ No sé<br>☐ Otro<br>((Marque todos los que apliquen))
Si escogió otro motivo por el cual no esta usando un método de contracepción, explique el motivo	__________________________________
Si escogió que tiene miedo de los efectos secundarios, ¿cuáles son esos efectos secundarios?	__________________________________
Desde que dio a luz, ¿ha vuelto a quedar embarazada?	◯ Sí<br>◯ No<br>◯ No sé

¿Qué pasó con el embarazo?

- ◯ Aborto espontáneo
- ◯ Terminación del embarazo
- ◯ Actualmente, estoy embarazada

ENFERMERA: SI LA PACIENTE ESTÁ ACTUALMETNE EMBARAZADA, DEBERÍA ADVERTIRLE QUE PARE SU MÉTODO ANTICONCEPTIVO SI LO SIGUE USANDO Y QUE SE INSCRIBA EN MADRES SANAS.

Llamada de Seguimiento- 3 Meses (Grupo de Control)

¿Ha empezado un método anticonceptivo desde que dió a luz?	◯ Si<br>◯ No
¿Qué método está utilizando?	☐ Abstinencia<br>☐ Método natural de familia/método rítmico<br>☐ Amenorrea de la lactancia/ dar de mamar<br>☐ Condones<br>☐ Pastillas<br>☐ Inyección<br>☐ Implante<br>☐ Dispositivo intrauterino (DIU)<br>☐ Operación/Ligadura<br>☐ Mi esposo se hizo la vasectomía<br>((Marque todos los que apliquen))
¿Qué tan satisfecha esta con este método?	◯ Muy satisfecha<br>◯ Un poco satisfecha<br>◯ Un poco insatisfecha<br>◯ Muy insatisfecha
¿Por qué esta insatisfacción con este método?	☐ El medicamento causa efectos secundarios<br>☐ Es muy cara<br>☐ Es muy difícil de obtener<br>☐ No está siempre disponible<br>☐ Otro
Si escogió otro, explique	______________________________
Si escogió que la medicación causó efectos secundarios, ¿cuáles son esos efectos secundarios?	______________________________
Si "no", ¿por qué?	☐ Quiero quedar embarazada<br>☐ No tengo una pareja entonces no necesito un método anticonceptivo.<br>☐ Mi pareja no esta de acuerdo que use un método anticonceptivo.<br>☐ Tengo miedo de los efectos secundarios<br>☐ No quiero empezar un método anticonceptivo porque no voy a poder continuarlo por problemas de tiempo, costo, y transporte.<br>☐ No sé<br>☐ Otro<br>((Marque todos los que apliquen))
Si escogió otro motivo por el cual no esta usando un método de contracepción, explique el motivo	______________________________
Si escogió que tiene miedo de los efectos secundarios, ¿cuáles son esos efectos secundarios?	______________________________
Desde que dio a luz, ¿ha vuelto a quedar embarazada?	◯ Sí<br>◯ No<br>◯ No sé

¿Qué pasó con el embarazo?

- ◯ Aborto espontáneo
- ◯ Terminación del embarazo
- ◯ Actualmente, estoy embarazada

ENFERMERA: SI LA PACIENTE ESTÁ ACTUALMETNE EMBARAZADA, DEBERÍA ADVERTIRLE QUE PARE SU MÉTODO ANTICONCEPTIVO SI LO SIGUE USANDO Y QUE SE INSCRIBA EN MADRES SANAS.

Llamada de Seguimiento- 12 Meses

Actualmente, ¿está usando un método anticonceptivo?	◯ Si<br>◯ No
¿Qué método está utilizando?	☐ Abstinencia<br>☐ Método natural de familia/método rítmico<br>☐ Amenorrea de la lactancia/ dar de mamar<br>☐ Condones<br>☐ Pastillas<br>☐ Inyección<br>☐ Implante<br>☐ Dispositivo intrauterino (DIU)<br>☐ Operación/Ligadura<br>☐ Mi esposo se hizo la vasectomía<br>((Marque todos los que apliquen))
¿Qué tan satisfecha esta con este método?	◯ Muy satisfecha<br>◯ Un poco satisfecha<br>◯ Un poco insatisfecha<br>◯ Muy insatisfecha
Por favor, discúlpese por esta insatisfacción y pregunte ¿por qué?	☐ El medicamento causa efectos secundarios<br>☐ Es muy cara<br>☐ Es muy difícil de obtener<br>☐ No está siempre disponible<br>☐ Otro<br>((Marque todos los que apliquen))
Si escogió otro, explique	______________________________
Si escogió que el medicamento causó efectos secundarios, ¿cuáles son esos efectos secundarios?	______________________________
Si "no", ¿por qué?	☐ Quiero quedar embarazada<br>☐ No tengo una pareja entonces no necesito un método anticonceptivo.<br>☐ Mi pareja no esta de acuerdo que use un método anticonceptivo.<br>☐ Tengo miedo de los efectos secundarios<br>☐ No quiero empezar un método anticonceptivo porque no voy a poder continuarlo por problemas de tiempo, costo, y transporte.<br>☐ No sé<br>☐ Otro<br>((Marque todos los que apliquen))
Si escogió otro motivo por el cual no esta usando un método de contracepción, explique el motivo	______________________________
Si escogió que tiene miedo de los efectos secundarios, ¿cuáles son esos efectos secundarios?	______________________________
Desde que dio a luz, ¿ha vuelto a quedar embarazada?	◯ Sí<br>◯ No<br>◯ No sé

¿Qué pasó con el embarazo?	◯ Aborto espontáneo<br>◯ Terminación del embarazo<br>◯ Actualmente, estoy embarazada

ENFERMERA: SI LA PACIENTE ESTÁ ACTUALMETNE EMBARAZADA, DEBERÍA ADVERTIRLE QUE PARE SU MÉTODO ANTICONCEPTIVO SI LO SIGUE USANDO Y QUE SE INSCRIBA EN MADRES SANAS.

Forma de Extracción de Implante

¿Por qué quieres eliminar(quitarte) tu implante?	☐ Quiero quedar embarazada<br>☐ No tengo una pareja entonces no necesito un método anticonceptivo.<br>☐ Mi pareja no está de acuerdo que use un método anticonceptivo.<br>☐ Tengo efectos secundarios<br>☐ No sé<br>☐ Otro
¿Cuáles son estos efectos secundarios?	______________________________
